# Perceptions and Experiences of Internet-Based Testing for Sexually Transmitted Infections: Systematic Review and Synthesis of Qualitative Research

**DOI:** 10.2196/17667

**Published:** 2020-08-26

**Authors:** Tommer Spence, Inès Kander, Julia Walsh, Frances Griffiths, Jonathan Ross

**Affiliations:** 1 Division of Health Sciences Warwick Medical School University of Warwick Coventry United Kingdom; 2 Whittall Street Clinic University Hospitals Birmingham National Health Service Foundation Trust Birmingham United Kingdom

**Keywords:** sexually transmitted infections, self-sampling, screening, testing, internet, digital health, eHealth, qualitative research, thematic synthesis

## Abstract

**Background:**

Internet-based testing for sexually transmitted infections (STIs) allows asymptomatic individuals to order a self-sampling kit online and receive their results electronically, reducing the need to attend a clinic unless for treatment. This approach has become increasingly common; however, there is evidence that barriers exist to accessing it, particularly among some high-risk populations. We review the qualitative evidence on this topic, as qualitative research is well-placed to identify the complex influences that relate to accessing testing.

**Objective:**

This paper aims to explore perceptions and experiences of internet-based testing for STIs among users and potential users.

**Methods:**

Searches were run through 5 electronic databases (CINAHL, EMBASE, MEDLINE, PsycINFO, and Web of Science) to identify peer-reviewed studies published between 2005 and 2018. Search terms were drawn from 4 categories: STIs, testing or screening, digital health, and qualitative methods. Included studies were conducted in high-income countries and explored patient perceptions or experiences of internet-based testing, and data underwent thematic synthesis.

**Results:**

A total of 11 studies from the 1735 studies identified in the initial search were included in the review. The synthesis identified that internet-based testing is viewed widely as being acceptable and is preferred over clinic testing by many individuals due to perceived convenience and anonymity. However, a number of studies identified concerns relating to test accuracy and lack of communication with practitioners, particularly when receiving results. There was a lack of consensus on preferred media for results delivery, although convenience and confidentiality were again strong influencing factors. The majority of included studies were limited by the fact that they researched hypothetical services.

**Conclusions:**

Internet-based testing providers may benefit from emphasizing this testing’s comparative convenience and privacy compared with face-to-face testing in order to improve uptake, as well as alleviating concerns about the self-sampling process. There is a clear need for further research exploring in depth the perceptions and experiences of people who have accessed internet-based testing and for research on internet-based testing that explicitly gathers the views of populations that are at high risk of STIs.

**Trial Registration:**

PROSPERO CRD42019146938; https://www.crd.york.ac.uk/prospero/display_record.php?RecordID=146938

## Introduction

### Background

Sexually transmitted infections (STIs) are a serious public health problem, with the incidence of many infections rising rapidly [1-3]. In England, syphilis diagnoses have risen 126% in the past 5 years, and gonorrhea diagnoses rose 26% in a year from 2017 to 2018 [3,4]. This statistic is of particular concern, given the increasing risk of antibiotic-resistant gonorrhea [3,5].

One of the challenges in preventing the spread of STIs is that they frequently remain asymptomatic [6,7]. This allows them to be spread unknowingly and increases the likelihood of developing complications such as pelvic inflammatory disease and infertility from chlamydia and gonorrhea and damage to the heart, bones, and central nervous system from syphilis [5,8,9].

Screening for STIs is therefore crucial in tackling their impact, ensuring that people are treated soon after infection, and reducing the risk of passing the infection onto others. It is well established, however, that numerous barriers exist to accessing testing, including stigma, aversion to the sampling process, or the time and travel required to access clinics [10-12]. This contributes to low uptake of testing, identified as an obstacle to reducing STI prevalence in a number of countries, including Australia, England, France, and the United States [1,3,13,14].

One new method to improve access to and uptake of STI screening is internet-based testing. Its use has grown rapidly in recent years, and it now accounts for over 17% of chlamydia tests undertaken by young people in England [3]. This figure is likely to continue rising, in part due to increased provision, as the cost effectiveness of internet-based testing mitigates considerable cuts to the budgets of sexual health services seen since 2013 [15]. Although variations exist between internet-based testing services, they almost all involve users ordering a self-sampling kit online, which they then return to a laboratory for testing before receiving their results remotely [16,17]. Common media for results delivery include SMS text messaging, email, phone, mail, and websites [16].

Although internet-based testing appears to address many of the barriers users face in accessing traditional face-to-face testing, there is a lack of conclusive data on how it is perceived or experienced. The existing systematic reviews focusing exclusively on self-sampling for STIs have found it to be acceptable, but these were not limited to internet-based testing and only included the views of people who had already accessed self-sampling [18-20]. Other reviews of attitudes towards STI testing have found that participants identified waiting times and clinic opening hours as examples of barriers to accessing face-to-face testing, but again these studies did not focus exclusively on internet-based testing and reported only limited data on self-sampling [21-23]. This review seeks to fill this gap and develop the understanding of how internet-based testing specifically is perceived and experienced. It focuses on qualitative research, as this approach is uniquely well placed to aid nuanced analysis of people’s engagement with sexual health services [24].

### Review Question

This review aims to answer the following question: What are the perceptions and experiences of internet-based testing for STIs among users and potential users?

## Methods

The review protocol was registered on PROSPERO during the review process (identification number CRD42019146938) [25].

### Search Strategy

The search used 5 electronic databases that specialize in health research: MEDLINE, EMBASE, CINAHL, Web of Science, and PsycINFO. The search terms were developed through experimentation with the support of a specialist librarian, using a population, intervention, context, and outcome model adapted for qualitative research [26,27]. This resulted in the following 4 search term categories: (1) Population: individuals with or at risk of STIs (eg, chlamydia); (2) Intervention: testing or screening for STIs (eg, test); (3) Context: online (eg, internet); and (4) Outcome: qualitative perception or experience (eg, interview).

An example list of search terms is included in Multimedia Appendix 1.

The search period spanned from January 1, 2005, to December 31, 2018. We chose 2005 because this was when internet-based testing emerged, and fewer than half of UK households had access to the internet prior to this period [28,29]. The search was limited to studies published in English, as there were insufficient resources available to arrange translation.

### Eligibility

Studies were eligible for inclusion if they (1) reported user (or prospective user) perceptions or experiences of any aspect of internet-based testing for STIs, either hypothetically or in practice, and how this affected whether users might access it; (2) collected the relevant data using qualitative methods, including the qualitative component of mixed methods research and free text responses to questionnaires; (3) were published in English in peer-reviewed academic journals between 2005 and 2018; and (4) collected data in countries defined as high-income countries by the World Bank.

Inclusion was limited to high-income countries due to their similar STI profiles, health care infrastructure, and rates of internet access [30,31]. Self-sampling for HPV was not included within the scope of this review, as it is normally conducted in the context of cancer screening and is not usually a component of STI screening [32].

### Screening

The search results from each database were combined and duplicates were removed. Initially, all studies were screened via their titles and abstracts to determine eligibility, with potentially eligible studies subsequently being read in full. These stages of the screening process were both undertaken in full by the lead reviewer (TS), with a second reviewer (IK) screening a random 20% (267/1332 for title and abstract screening; 16/79 for full-text screening) of studies at each stage to determine interrater reliability. Any disagreements were resolved through discussion, and a third reviewer was brought in if consensus could not be reached. Reviewers always erred on the side of inclusion to ensure all relevant data were identified.

### Quality Assessment

The quality of the studies was critically appraised using the Critical Appraisal Skills Programme checklist for qualitative research [33]. The checklist provides a holistic overview of the rigor of research, and any studies found to be methodologically weak were planned to be included in a sensitivity analysis once the synthesis was complete.

### Data Extraction and Synthesis

The included studies underwent thematic synthesis following an adapted version of a framework developed by Thomas and Harden [34] for qualitative systematic reviews. The results section of each study was uploaded to NVivo (QSR International), with each line of text that addressed the research question being coded according to its meaning and content. All relevant data were coded at least once, and once this process was complete, codes were grouped into descriptive themes that captured the meaning of multiple initial codes. These were in turn developed into broader analytical themes, which go beyond the findings of the included studies. The lead reviewer (TS) undertook all analysis, with support and interrater reliability being provided on 20% of included studies by a second reviewer (JW) and feedback being provided from the lead reviewer’s supervisors (FG and JR).

## Results

### Search

**Figure 1:**
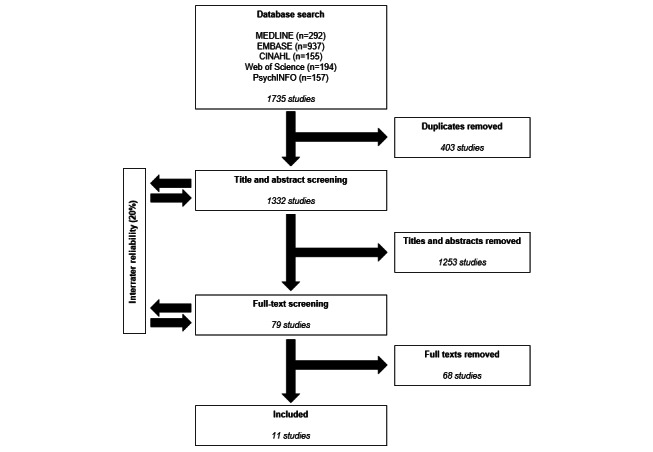
Summary of literature search process.

A total of 11 studies met the inclusion criteria for the review. The initial search identified 1735 studies for screening, which reduced to 1332 studies once duplicates were removed. The search and screening process is outlined in Figure 1, along with the number of studies removed at each stage in the process.

The initial rate of agreement between reviewers was 96.2% for title and abstract screening and 93.3% for full-text screening.

### Study Characteristics

An overview of the included studies is provided in Table 1. All were published between 2006 and 2018. One was conducted in Australia, with the remainder split equally between the United Kingdom and United States.

**Table 1:** Characteristics of included studies.

Lead author	Year	Country	Aim	Participants	Study size	Average age (age range)	Actual or potential users	Methods
Ahmed-Little [35]	2016	United Kingdom	To explore attitudes of participants towards a pilot internet-based HIV-testing program	People who provided a free-text response within a questionnaire	756	Mean 22.6 (16-72)	Actual	Free text space in a feedback questionnaire, thematically analyzed
Baraitser [17]	2015	United Kingdom	To obtain stakeholder input on a theory of change for online sexual health services	Potential service users sampled to include men who have sex with men, young people, and ethnic minorities	4	—^a^	Potential	Interviews, analyzed using a framework approach
Friedman [36]	2013	United States	To explore young women’s technology use, preferences for STD^b^-testing venues, attitudes toward nontraditional venues, and acceptability of different test results delivery methods	Women (35% Black, 34% Hispanic, 31% White), recruited through market research firms	80	— (15-25)	Potential	Ethnographic semistructured interviews, thematically analyzed
Gaydos [37]	2006	United States	To inform the design of an effective educational website that facilitates self-sampling and is appealing to women	Women (57% Black, 2% Hispanic, 43% White), recruited via educational institutions	42	— (14-49)	Potential	Focus groups, reported
Gibbs [38]	2018	United Kingdom	To evaluate the results component of a pilot online sexual health service	People purposively sampled from users of the pilot service	36	— (18-35)	Actual	In-depth interviews, analyzed using a framework approach
Lorimer [39]	2013	United Kingdom	To inform the design of an internet-based approach to chlamydia screening targeting young men	Men recruited from the community	60	— (16-24)	Potential	Focus groups, analyzed using a framework approach
Roth [40]	2011	United States	To explore preferences for accessing STI^c^-screening services among men	Men (55% Black, 14% Hispanic, 31% White) recruited in a sexual health clinic	29	Median 34 (19-60)	Potential	Interviews and focus groups, thematically analyzed
Stahlman [41]	2015	United States	To explore attitudes towards potential interventions to increase testing and reduce transmission among MSM^d^ with repeat syphilis infection	MSM (16% Black, 32% Hispanic, 53% White; 68% HIV+), recruited via local government public health database	19	Mean 38 (21-54)	Potential	Semistructured interviews, analyzed using grounded theory and thematically via axial coding
Tobin [42]	2018	United States	To assess the acceptability and feasibility of a program to train young Black MSM to use and promote HIV and STI home testing to their social network	Young Black MSM (2 self-reported HIV+) recruited from the community	15	Mean 26.2 (—)	Potential	2 in-depth structured interviews, 1 week apart, reviewed for range, consensus, and divergence of responses
Tomnay [43]	2014	Australia	To examine rural young people’s perceptions of barriers and facilitators to using face-to-face and online sexual health testing and treatment	Young people recruited from the community	50	— (16-25)	Potential	Focus groups, thematically analyzed
Wayal [44]	2011	United Kingdom	To inform the development of a service offering home sampling kits for STI/HIV	MSM (4% Black, 92% White, 4% Asian; 17% HIV+), recruited from a sexual health clinic	24	Median 39 (22-68)	Potential	Focus groups, analyzed using a framework approach

^a^Not available.

^b^STD: sexually transmitted disease.

^c^STI: sexually transmitted infection.

^d^MSM: men who have sex with men.

Of the 11 studies included, 2 studies reported the experience of users who had accessed internet-based testing, with the remaining 9 exploring perceptions among potential users of hypothetical services. Of these, 2 studies explored the views of women, 2 explored the views of men who have sex with men (MSM), and 2 explored the views of men whose sexual orientation was unspecified. The remaining 3 studies explored the views of both men and women.

Young people aged younger than 30 years were exclusively recruited to 4 of the studies, including one of the studies that explored the views of women, one that explored the views of men, and one that explored the views of MSM. This latter study also had exclusively Black participants; another 4 studies reported over 45% of participants as people of color.

### Critical Appraisal

**Figure 2:**
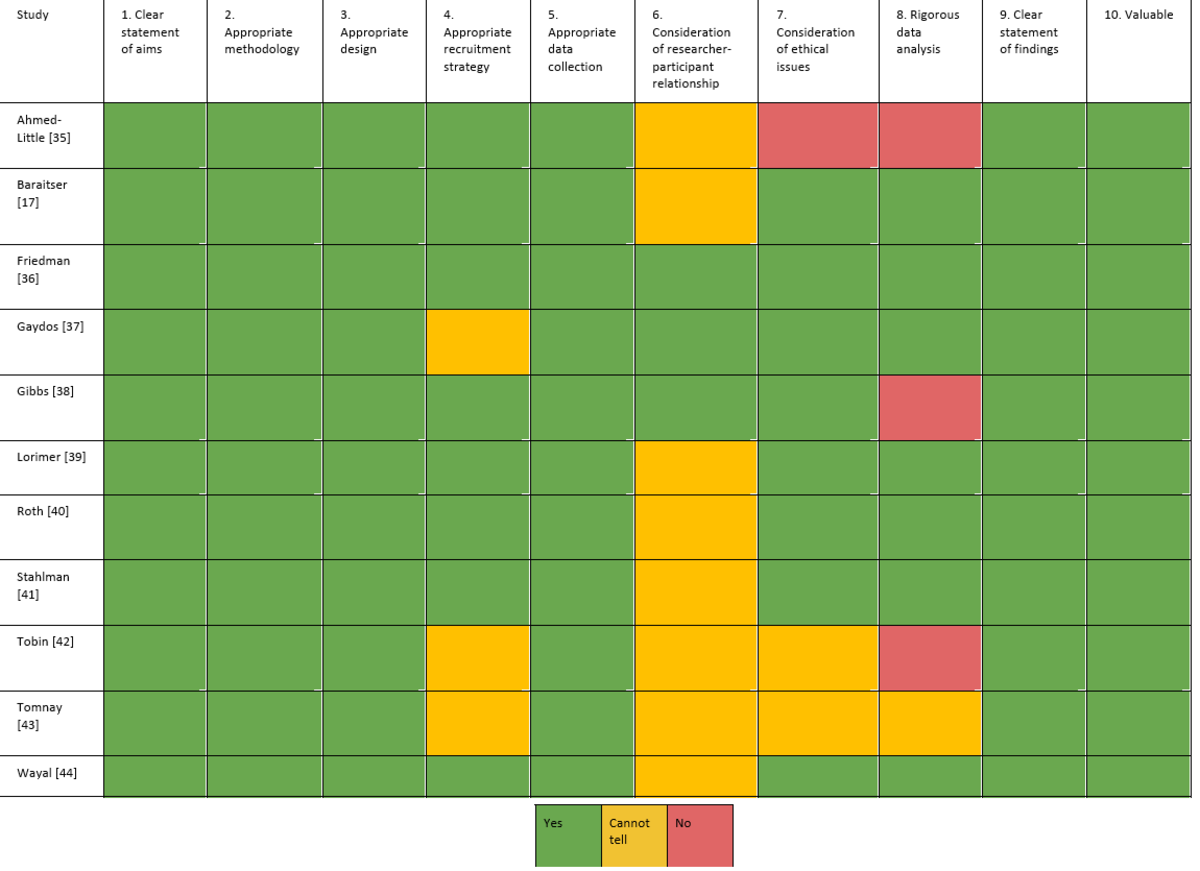
Critical appraisal of included studies according to the Critical Appraisal Skills Programme.

The results of the critical appraisal are outlined in Figure 2. All but 3 [35,42,43] of the studies met at least 9 of the 10 appraisal criteria, with the remainder [17,36-41,44] meeting at least 6. All of the studies were deemed valuable, had clear aims and statements of findings, and had appropriate methodologies, study designs, and data collection methods. However, most did not provide enough information to determine whether they met all of the criteria, with only 3 [36-38] reporting sufficient consideration of the researcher-participant relationship. Separately, 3 studies [35,38,42] described their analysis with limited detail, which meant it was not clear how themes were derived from the data and the methods were not replicable.

### Synthesis

A total of 12 themes were identified, which were organized into 4 categories. These are outlined in Table 2.

**Table 2:** Summary of themes.

Categories	Themes
1. Positive aspects of internet-based testing	1.1 Internet-based testing is acceptable 1.2 Attractive due to convenience 1.3 Attractive due to the stigma associated with face-to-face testing 1.4 Avoids undesirable aspects of face-to-face testing 1.5 Improves accessibility of STI^a^ testing
2. Negative aspects of internet-based testing	2.1 Loss of positive aspects of face-to-face testing 2.2 Concerns about self-sampling processes 2.3 Privacy concerns with internet-based testing
3. Positive aspects of remote delivery of results	3.1 Remote delivery of results is acceptable 3.2 Convenience drives preference of results medium
4. Negative aspects of remote delivery of results	4.1 Concern about interception 4.2 Concern over well-being

^a^STI: sexually transmitted infection.

#### Category 1: Positive Aspects of Internet-Based Testing

##### Internet-Based Testing Is Acceptable

There was a broad yet incomplete consensus that internet-based testing was acceptable, with around half of studies explicitly reporting that participants were open or positive towards using it [35,37,39,41,42]. Ahmed-Little et al [35], for example, reported:

There was overwhelming support that this method of testing offered ease and was considered acceptable.

A minority of studies did report uncertainty or negativity among some potential users of hypothetical internet-based testing services, however [36,37,39]. Friedman and Bloodgood [36] reported that overall:

Participants were slightly more negative than positive about the option of ordering [a sexually transmitted disease] test from a website.

##### Attractive Due to Convenience

The perceived convenience of internet-based testing was a prominent theme, appearing in almost all of the included studies [35-37,39-44]. Approximately half of them identified that internet-based testing appealed to participants because it meant they would not have to take time out of their day to get tested or go to the effort of travelling to a clinic [35,37,39,40,43]:

You just go in there, ﬁnd the information that you need and don’t have to worry about travelling, getting gas, whatever, so...it’s quick and easy.

40

The convenience of not having to make or wait for an appointment also enhanced the appeal of internet-based testing among participants, especially those who had accessed it:

I think this is a great service as I have tried to do this through my doctor and will have to wait 3 weeks for an appointment.

35

##### Attractive Due to the Stigma Associated With Face-to-Face Testing

Internet-based testing appealed to many participants due to the perceived anonymity, confidentiality, or privacy it offers compared with face-to-face testing. This theme appeared in most of the studies included in the review and applied to women and those who had accessed internet-based testing in particular [17,35-37,39-42,44]. Many participants felt embarrassed, anxious, or ashamed about the prospect of others seeing them at a clinic or finding out they had attended one:

If you can do it all remotely and without anybody knowing or seeing you waiting outside a sexual health clinic and going, “Oh, what are you doing here?” then I think it’s going to be absolutely brilliant.

17

##### Avoids Undesirable Aspects of Face-to-Face Testing

Around a third of the included studies reported that participants were attracted to internet-based testing because it allowed them to avoid specific aspects of face-to-face testing that they disliked, a finding that was prominent among those who had accessed internet-based services [17,35-37]. The most prevalent of these aspects was interacting with clinic staff, a negative prospect for many participants, particularly women. Friedman and Bloodgood [36] reported one participant stating that they liked the idea of internet-based testing, as it meant:

I don’t have to…have this long talk with a professional about sexual education.

Ahmed-Little et al [35] and Gaydos et al [37] both reported participants stating that internet-based testing would be appealing for users who experience anxiety about interacting with a health care professional. These same two studies also identified that some participants preferred internet-based testing to face-to-face testing, as it was more comfortable and allowed them to avoid sampling methods they were averse to, such as venipuncture:

I am terriﬁed of needles so this small lancet is much easier I would rather prick my ﬁnger than have a needle in my arm.

35

##### Improves Accessibility of STI Testing

Almost half of the included studies reported that participants felt internet-based testing would improve access to testing for STIs, as it would allow them to overcome barriers such as not being able to afford face-to-face testing, not having easy access to it, or feeling averse to using it. This finding emerged strongly among participants who had accessed internet-based testing and was seen as particularly advantageous for young people, with Gaydos et al [37] reporting one participant stating:

It is always good to have several ways to get tested at no cost or low cost especially for teens.

There were a small number of studies, however, that recorded concerns about potential barriers to internet-based testing, such as cost or a lack of internet access. Tomnay et al [43], for example, found that:

An important consideration for all groups was that online STI testing is a free service…[R]esearchers were asked by participants about the cost of using a website for online testing with questions such as ‘so this is all free?’ and including speciﬁc questions such as ‘would they [referring to the website] pay the postage to send it back?’ in reference to returning the testing kit.

#### Category 2: Negative Aspects of Internet-Based Testing

##### Loss of Positive Aspects of Face-to-Face Testing

Over half of the included studies found that some participants were dissuaded from using internet-based testing, as they felt it was lacking important aspects of face-to-face testing [36,37,39-41,44]. One common example was the opportunity to speak with a health care provider about health holistically. This attitude appeared to be more widely held by men, as evidenced by Roth et al [40], who reported a participant saying:

[I want to talk to a professional] when I’m thinking if I need to take a test. You know, what was the probability I was infected? What do I need to know about how transmission occurs? Are there any studies or statistical data that correspond with my particular case that would give me a clue in on how worried about it do I really need to be?

There was an implication in some of the studies reporting this finding that this view was predominantly held by participants who were familiar and comfortable with the provider they would use to get tested for STIs and that it was not found among participants who had accessed internet-based testing [35,39,41].

##### Concerns About Self-Sampling Processes

Over half of the included studies identified concerns among participants about the self-sampling process [35-37,41,42,44]. One aspect of this was the prospect of challenges or discomfort caused by self-sampling, as reported by one participant in Tobin et al [42]:

I showed them the packet. And the first question they asked was ‘Is it painful?’ ... Once I told them it was painless they were a little more interested.

Tobin et al [42] also identified the other prevalent concern about self-sampling: it may be inaccurate or unreliable. They report one participant stating:

If it is not 100% accurate they [peers] would probably prefer to go to a clinic. Even if I am telling them, they might not feel they know enough about the at-home test and might think it is better to go to a clinic.

42

Another aspect of the process that a number of studies identified as a concern was the return of samples, which a number of participants felt may compromise the test in some way [36,40,44]. Roth et al [40] reported:

The top ranked method for sample return was in person, even among individuals who preferred self-sampling … Recurrent themes for personally returning the sample to the clinic included the possibility of sample misidentiﬁcation, the possibility of loss of conﬁdentiality, mistrust of the postal system and immediate access to treatment.

Uncertainty around the self-sampling appeared to be more prevalent among men and in studies with ethnically diverse participants, although such concerns were not ubiquitous in these populations.

##### Privacy Concerns With Internet-Based Testing

A number of studies researching perceptions of hypothetical services identified privacy concerns among participants relating to internet-based testing [37,39-41,43]. These largely centered around obtaining or returning self-sampling kits, with young people in particular expressing concern about their parents finding a kit in the mail. Tomnay et al [43] reported a teenaged participant stating:

You don’t want your parents to know about it, every day you’re going to be the first one to that mailbox checking to see whether it’s there.

Gaydos et al [37] and Roth et al [40] both reported concerns among participants about being seen collecting or returning a kit in a public location, a theme that appeared to be stronger in studies with ethnically diverse populations, while Stahlman et al [41] noted concern about data being collected by providers:

One participant noted that he would not be comfortable submitting identifying information, such as his name, online.

#### Category 3: Positive Aspects of Remote Delivery of Results

##### Remote Delivery of Results Is Acceptable

The included studies that sought participant views on results delivery covered a wide range of media, including SMS, email, websites, and mail. A variety of options for phone delivery were also covered, including a phone call from a health practitioner, an answering machine message, and having to proactively call in to obtain results. The diversity of the media investigated means it is difficult to draw firm conclusions on any particular option, as opposing views were identified on almost every medium. Nevertheless, almost half of the included studies reported positive attitudes towards the delivery of results electronically. This included Gibbs et al [38], who explored users’ experiences of receiving results via a website and found that:

They welcomed the online results service, for the ability it gave them to log on when they felt ready.

Ahmed-Little et al [35]*,* the other study that explored participants’ experiences of using internet-based testing, also found participants to be positive about electronic results.

Results by phone were also found to be viewed positively in almost a third of studies [36,37,40,44]. The most mixed response was found towards the delivery of results by mail [36,37,40].

##### Convenience Drives Preference of Results Medium

Almost a third of studies identified that participants’ preferences for a results medium were frequently influenced by its perceived convenience [36,38,40]. Roth [40], for example, quoted a participant discussing SMS results:

I can just ﬂip my phone up real quick, even at work, and like okay, cool. And then I can just know what the results were and it’d just be nice. It’d be easy.

Friedman and Bloodgood [36], meanwhile, found the same motivation among participants who preferred email notification and an answering machine message on their phone.

#### Category 4: Negative Aspects of Remote Delivery of Results

##### Concern About Interception

Almost all of the studies that had data on attitudes towards results notification reported that one of users’ main concerns was interception, although this manifested itself in differing preferences [36-40]. Gaydos et al [37] explored attitudes towards a number of media and found concerns relating to all of them from some participants. One said of results via a website, for example:

Typing in a passcode for results on the internet is a good idea but most families use the same computer so you will have to be careful not to leave your passcode lying around.

37

Other participants shared concerns about mail being read by family members and calls being overheard. This theme appeared to be prominent among women and those who had accessed internet-based testing.

##### Concern Over Well-Being

Almost half of the included studies reported that attitudes towards results delivery were motivated in part by a concern for the well-being of users [36-38,40,42]. This was most frequently expressed through the concern that communication of results via electronic media would mean that users would not receive sufficient support or advice, particularly if the results were positive. This also appeared to be a commonly held attitude by women, with Friedman and Bloodgood [36] quoting one participant who had received results face-to-face:

It was nice to speak to somebody and for them to tell me ‘okay, this is what we did, this is the tests we ran and thank God, you’re negative.’ Again, just somebody to talk to so that if you had questions, you had somebody face to face to talk to.

A number of participants were also concerned that the use of certain media for results delivery may result in users being left unaware of a positive diagnosis, for example, if they had to proactively call in to be informed or needed to remember a password to access results online. One participant was quoted by Roth et al [40] as saying:

I don’t think email would be good because everybody gets junk mail they might just delete the email without even knowing.

In contrast, Gibbs et al [38] reported that participants appreciated being able to log on to a website and access their results whenever they felt mentally prepared to do so.

## Discussion

### Principal Results

This review and synthesis identified a wide range of perceptions and experiences of internet-based testing held by users and potential users. There was a clear finding that internet-based testing is attractive due to its convenience and the fact that it alleviates concerns around stigma associated with being tested in a clinic, and many participants were drawn to it because it allowed them to avoid elements of face-to-face testing that they disliked. However, there was also a concern among some participants relating to the privacy of internet-based testing, the self-sampling process, and the fact that internet-based testing would be missing positive aspects of face-to-face testing. There was no universally accepted medium for results delivery, but preference was largely motivated by perceptions of convenience and concerns over privacy. Overall, internet-based testing appears to be acceptable despite some reservations expressed about it.

### Strengths and Limitations

This review is the first attempt we are aware of to bring together qualitative data that relate to this growing medium of STI testing. The inclusion of data from 1115 participants—a substantial number for qualitative research—is a notable strength, as is the fact that the data were synthesized using a well-established and transparent method. All of the studies were assessed to be of satisfactory quality, imbuing confidence in the results, and the fact that all studies were undertaken in 3 countries with similar socioeconomic profiles enhances the generalizability of the results.

The synthesis was limited by the small number of studies eligible for inclusion, and the quantity of data varied widely between these studies. This meant that although there was a relatively strong consensus among included studies on most themes, it was difficult to draw definitive conclusions on subpopulations. Only 2 of the studies collected data from people who had actually used internet-based testing, for example, and one of these collected data only on their experience receiving results, which limited the distinctions that could be drawn between their findings and the findings of the 9 studies that collected data from potential users of hypothetical services. This similarly affected the findings on perceptions held by different sociodemographic populations, and the review may also have benefited from studies undertaken in a wider number of geographical settings and published in languages other than English. The included studies are also unlikely to have recruited people from vulnerable populations, such as homeless people or those with serious mental health issues, meaning these populations’ views may not have been captured in the review. The screening of studies for inclusion was limited, as only a proportion of results were screened by 2 authors, and although including studies published between 2005 and 2018 ensured that all relevant data were included, this relatively long time frame means that findings from some of the earlier studies may now be less relevant due to considerable changes in internet usage over this period.

### Comparison With Prior Work

Internet-based STI testing differs from many other digital health interventions (such as telehealth, patient portals, and remote monitoring), as it does not involve two-way communication with clinical staff and is designed as a one-off engagement with the health care system rather than part of the management of a long-term condition. This review offers the opportunity to explore whether users of STI-testing services interact with the service in the same way as those using other digital health interventions, and one notable difference is the fact that internet-based testing is highly associated with convenience. This contrasts with the findings of a review by O’Connor et al [45] about the factors affecting more general engagement with digital health interventions, which found that many people could be deterred from accessing interventions if they felt they lacked the time or energy to do so, which may be because such interventions are frequently targeted towards individuals who are expected to have more sustained engagement [46].

However, other findings were similar to those found for alternative digital interventions, including the finding that some participants had privacy concerns over electronically providing the personal data required for access and that other participants appreciated the anonymity offered by digital interactions over face-to-face ones [45,47]. This concern over the provision of personal data parallels evidence on engagement with digital media unrelated to health care, such as social media, which regularly forces users to weigh privacy concerns against perceived benefits of use [48,49]. The normalization of social media can frequently lead users to overlook their privacy concerns, however, and this is unlikely to apply in the same way when people access internet-based testing, given that it is less likely to feature as often in their day-to-day lives [48-50].

The stigma associated with sexual health also undoubtedly heightens privacy concerns held by users of internet-based testing [23]. The concerns over privacy and anonymity identified in this review simultaneously highlight the potential of digital interventions to overcome stigma as a barrier to accessing health care—in the context of both sexual health and other conditions—and the underresearched phenomenon of the role that stigma plays when using internet-based interventions [23,45,51,52]. Our finding that many people are concerned about aspects of the internet-based testing process that could allow others to know they had used sexual health services emphasizes the role providers have in mitigating that risk and suggests a need for further exploration of the role that stigma plays when individuals access, or consider accessing, internet-based health care. It is noteworthy that this finding was prominent in studies with ethnically diverse populations and that women were found to be particularly concerned about the stigma associated with clinics and interacting with clinic staff, as users of internet-based testing are disproportionately women and White [53-55].

The concern identified that internet-based testing may deprive users of important aspects of face-to-face testing is significant, particularly as internet-based testing is one of the few health interventions that allows users to have no direct contact with clinical staff. Although it did not appear to deter the majority of participants from the prospect of using internet-based testing, it aligns with evidence from other contexts that digital health care is seen as supplementary and that service users are willing to use it provided it does not replace face-to-face care [51]. This may also be the case for the delivery of test results remotely, a topic on which limited evidence exists and to which this review therefore makes a notable contribution [56]. Our finding that people who had used internet-based testing were satisfied to receive their results by SMS is in agreement with other research, which has found high levels of satisfaction among patients who have received results electronically [57]. That we found no consensus on preferred media for results delivery among potential users corresponds with conflicting data from other sexual health studies, suggesting that service users’ preferences when conceptualizing hypothetical services may not be an accurate predictor for what they find acceptable when they start using them [58-60].

### Recommendations for Practice

Providers of internet-based testing may wish to emphasize the approach’s comparative convenience and privacy compared with face-to-face testing in order to improve uptake, as these appear to be the most appealing aspects of the service. Uptake may also be improved through attempts to alleviate concerns around self-sampling, for example, by providing reassurance about discomfort and emphasizing that the sensitivity and specificity of self-sampling is comparable to samples obtained in a clinic. It may also be worthwhile for providers to consider patient confidentiality in results delivery, for example, by ensuring that text messages are worded so they could not inadvertently reveal a diagnosis in a phone notification, and to ensure that patients receive adequate signposting to support if results are delivered electronically.

### Conclusions

This study has identified a wide range of perceptions and experiences of internet-based testing by actual and potential users, including both positive and negative comparisons with clinic-based testing. There is a clear need for further qualitative research exploring in depth the experiences of people who have accessed internet-based testing, given the paucity of data on this, and for qualitative research on internet-based testing that explicitly gathers the views of populations that are at high risk of STIs or that have testing behaviors that require more in-depth understanding. There would also be value in further research on attitudes towards communicating results, given that no consensus could be found on a preferred medium.

## Appendix

Multimedia Appendix 1 Example electronic database search, MEDLINE.

## Abbreviations

MSM: men who have sex with men
STI: sexually transmitted infection
