# Supplementary material for: Perceptions and Experiences of Internet-Based Testing for Sexually Transmitted Infections: Systematic Review and Synthesis of Qualitative Research
Source: J Med Internet Res. 2020 Aug 26;22(8):e17667. doi: 10.2196/17667 (PMC7481875; doi:10.2196/17667)
Supplement: Multimedia Appendix 1 [file jmir_v22i8e17667_app1.docx]

**Appendix 1: Example electronic database search, MEDLINE**

| **#** | **Searches** |
| --- | --- |
| **1** | exp Reproductive Health/ or sexual health.mp. |
| **2** | exp Sexually Transmitted Diseases/ or sexually transmitted infection*.mp. or sexually transmitted disease*.mp. |
| **3** | exp Gonorrhea/ or exp Syphilis/ |
| **4** | chlamydia.mp. or exp Chlamydia/ or exp Chlamydia trachomatis/ or exp Chlamydia Infections/ |
| **5** | 1 or 2 or 3 or 4 |
| **6** | internet.mp. or exp INTERNET/ |
| **7** | exp ONLINE SYSTEMS/ or online.mp. |
| **8** | (home-sampl* or home-base*).mp. |
| **9** | technology.mp. |
| **10** | 6 or 7 or 8 or 9 |
| **11** | exp QUALITATIVE RESEARCH/ or qualitative.mp. |
| **12** | exp INTERVIEW/ or interview*.mp. |
| **13** | exp Focus Groups/ or focus group*.mp. |
| **14** | 11 or 12 or 13 |
| **15** | test*.mp. |
| **16** | screen*.mp. |
| **17** | 15 or 16 |
| **18** | 5 and 10 and 14 and 17 |
| **19** | limit 18 to (english language and yr="2005 - 2018") |
